# Transmission of *Calicophoron daubneyi* and *Fasciola hepatica* in Galicia (Spain): Temporal follow-up in the intermediate and definitive hosts

**DOI:** 10.1186/s13071-016-1892-8

**Published:** 2016-11-29

**Authors:** Javier Iglesias-Piñeiro, Marta González-Warleta, José Antonio Castro-Hermida, María Córdoba, Camino González-Lanza, Yolanda Manga-González, Mercedes Mezo

**Affiliations:** 1 Laboratorio de Parasitología, Centro de Investigaciones Agrarias de Mabegondo, Instituto Galego de Calidade Alimentaria-Xunta de Galicia, Carretera AC-542 de Betanzos a Mesón do Vento, Km 7, 15318 Abegondo (A Coruña), Spain; 2 Departamento de Zooloxía, Xenética e Antropoloxía Física, Facultade de Bioloxía, Campus Vida, Universidade de Santiago de Compostela, 15706 Santiago de Compostela (A Coruña), Spain; 3 Department of Crop and Soil Science, Oregon State University, Corvallis, OR 97331 USA; 4 Departamento de Sanidad Animal, Instituto de Ganadería de Montaña, Consejo Superior de Investigaciones Científicas (CSIC)-ULE, 24346 Grulleros (León), Spain

**Keywords:** *Calicophoron daubneyi*, *Fasciola hepatica*, Snail hosts, *Galba truncatula*, Seasonal trends, GAM

## Abstract

**Background:**

Paramphistomosis caused by *Calicophoron daubneyi* and fasciolosis caused by *Fasciola hepatica* are common parasitic diseases of livestock animals. Transmission of the diseases depends on the presence of intermediate hosts, i.e. freshwater gastropods such as lymnaeids. We carried out a 2-year-long study of the dynamics of the snail population acting as the intermediate host for these parasites, considering the population structure in terms of size/age and infection status. In addition, we determined the kinetics of trematode egg excretion in grazing cows. Generalized Additive Models (GAMs) were used to analyze the associations between different response variables and snail size, sampling month and weather-related variables.

**Results:**

Of the molluscan species examined, *Galba truncatula*, *Radix peregra*, *Anisus* (*Anisus*) *leucostoma* and *Pisidium casertanum* (*n* = 2802), only *G. truncatula* was infected with *C. daubneyi* or *F. hepatica*, at prevalence rates of 8.2% and 4.4% respectively. The probability of infection with *C. daubneyi* or *F. hepatica* was linearly related to snail size, although in different ways (negative for *C. daubneyi* and positive for *F. hepatica*). The total snail population increased in winter, when specimens of all size classes were found. Infected snails were more abundant during spring-autumn. Mature cercariae of both parasites were found in most seasons. In the statistical models, the sampling month accounted for a high percentage (71.9–78.2%) of the observed variability in snail abundance. The inclusion of climatic variables in the models moderately increased the percentage of deviance explained (77.7–91.9%). Excretion of *C. daubneyi* eggs in cow faeces was always higher than that of *F. hepatica* eggs.

**Conclusions:**

Particular care should be taken to prevent pastures and the surrounding environment being contaminated with parasite eggs during winter-spring, when the number of snails susceptible to miracidial infections is maximal. This is therefore the optimal time for treating grazing animals. Nevertheless, control of trematodosis based only on chemotherapy is difficult in an area such as the study area, where environmental factors favour the regular appearance of snail populations harbouring mature cercariae.

**Electronic supplementary material:**

The online version of this article (doi:10.1186/s13071-016-1892-8) contains supplementary material, which is available to authorized users.

## Background

Paramphistomosis caused by *Calicophoron daubneyi* (Dinnik, 1962) and fasciolosis caused by *Fasciola hepatica* Linnaeus, 1758 are among the most common parasitic infections of livestock animals. These infections appear concurrently on many farms. Fasciolosis has been recognized for many years as a severe pathological entity with detrimental effects on animal welfare and production performance. On the contrary, paramphistomosis has traditionally been considered of no medical significance, at least when livestock animals are maintained in good nutritional and health status, as is usual in Europe. However, this situation seems to be changing, since cases of serious illness caused by paramphistomes have been described in France [1] and the UK [2–4]. Increased prevalence of both infections has also been reported in various European countries [5–8]. The higher rate of prevalence may reflect an increased risk of infection, possibly triggered by changes in climatic conditions favouring higher transmission rates [9] and also a failure of the control measures applied, which are often limited to treatment of livestock with anthelmintics. A sustained reduction in infection rates can only be achieved if early reinfection of treated animals is prevented. This can only be done after identification of the spatio-temporal fluctuations in the risk of transmission of infection.

Transmission of the trematodes *C. daubneyi* and *F. hepatica* is associated with the presence of freshwater gastropods that act as the intermediate hosts (IHs) within which the parasites multiply. The amplifying efficiency of the intra-molluscan cycle determines the number of metacercariae produced and the associated risk of infection in grazing animals. The parasites do not multiply within the definitive hosts, and the magnitude of the burden in livestock, which in turn determines the morbidity of infection, depends directly on the number of metacercariae ingested. Information about the gastropod populations acting as IHs is required for a good understanding of the epidemiology of paramphistomosis and fasciolosis. Such information would also help minimize transmission through actions aimed at disrupting the life-cycle of the parasites.

Recent reports on the spatial distribution of paramphistomosis and fasciolosis explain the geographical heterogeneity in the probability of infection of livestock as a consequence of environmental factors driving the life-cycle of both IHs and trematodes [10–13]. However, as environmental (both abiotic and biotic) factors vary significantly throughout the year, the risk of infection also varies, and the temporary fluctuations in this parameter must therefore also be taken into account in epidemiological studies.

Many studies have confirmed that *Galba* (*Galba*) *truncatula* (Müller, 1774) is the main transmitter of *F. hepatica* within Europe [14–16], although some recent data show that other lymnaeid snail species can participate in the life-cycle [17, 18]. Although the snail *G. truncatula* can act as an IH for *C. daubneyi* [18–20], the involvement of other gastropod species in the life-cycle of this parasite has scarcely been studied [21]. In view of the key role of lymnaeid snails in the life-cycle of trematodes of medical and veterinary importance, present knowledge of the dynamics of snail populations and parasite-host interactions is insufficient.

In this study, we addressed some key aspects of the transmission of *C. daubneyi* and *F. hepatica* in a region where the infections caused by both trematodes are endemic. We first assessed whether the different gastropod species co-existing in the habitat act as IHs for *C. daubneyi* and *F. hepatica*. We then studied the dynamics of the snail population (*G. truncatula*) acting as IH for both parasites, over a period of two years, taking into account the population structure in terms of size/age and infection status. We also determined the kinetics of trematode egg excretion in cows that had access to the stream where the snails were sampled. Finally, we used Generalized Additive Models (GAMs) to analyze the associations between different factors (snail size, sampling month and weather-related variables) and the response variables studied.

## Methods

### Sampling

The study was carried out on a dairy cattle farm (43°4′N, 8°3′W) in Galicia (north-west Spain). The climate in the region is classified as temperate maritime, with an average annual temperature of 11.6 °C and average annual cumulative rainfall of 1065 mm [22]. Infections caused by both *F. hepatica* and *C. daubneyi* had previously been diagnosed on the farm *via* necropsy of some cows in the slaughterhouse [13]. On our first visit to the farm, we were shown the fields where the cows were pastured. We observed some snails (possible intermediate hosts for both parasites) in a narrow (<1 m), shallow (<50 cm), slow-flowing stream where the animals had free access for drinking. The cows grazed all year round and were never treated with flukicides. The farm was selected for study because of these conditions.

Throughout two consecutive years (between July 2007 and June 2009), we made monthly visits to the farm for simultaneous sampling of cows (definitive hosts) and snails (potential intermediate hosts). During each visit, all cows (*n* = 34) were sampled individually and faeces were removed directly from the rectum. Systematic sampling of snails was always carried out by the same three trained researchers, at the same time of day (between 11:00 h and 13:00 h) along the same stretch of the stream (150 m long), including the stream bed and strips of pasture (50 cm wide) on both sides of the stream. Information about the prevalence of natural infections caused by *F. hepatica* in the intermediate hosts in Spain is scarce, and there are no data available about natural infections caused by *C. daubneyi*. Thus, assuming a prevalence rate of infection of 50% (error margin 10% and confidence interval 95%), the minimum size of sample required was estimated to be 95 snails. Each collector searched 10 sites (about 5 m apart from each other) for 5 min per site and used soft forceps to pick up all gastropods seen by the naked eye. When fewer than 95 molluscs were collected, the sampling was repeated. With a view to validating the results obtained on this farm, a larger sample of freshwater gastropods (*n* = 1097) was randomly collected on other cattle farms with confirmed cases of paramphistomosis and fasciolosis.

### Parasitological techniques

The faecal samples were analyzed using a quantitative sedimentation technique. Briefly, 10 g of faeces was added to about 100 ml of tap water and mixed thoroughly until obtaining a homogeneous suspension. Glass beads were added to the mixture to help break up the material. The resulting suspension was then passed through a 150 μm sieve. The filtered material was transferred to a 500 ml conical flask for sedimentation (3 times each for 20 min). The final sediment was transferred to a tube and water was added until a volume of 10 ml. After homogenization, a 1 ml aliquot (containing 1 g of faeces) was removed and examined under the microscope (100×) to count the *Calicophoron* and *Fasciola* eggs. The results were expressed as number of eggs (of each type) per gram of faeces (epg).

The molluscs collected were classified on the basis of external morphological characteristics. Some representative specimens of each species were preserved in 70% ethanol for further accurate taxonomic classification.

Helminthological examinations were carried out in vivo. Each snail was measured (shell height from the apex to anterior margin) and the body was then extracted and dissected under a stereomicroscope to search for signs of trematode infection. When trematode larvae were detected, samples were subsequently examined under the microscope, at higher magnification, in order to confirm the species and determine the degree of development. The following 7 larval stages were recorded: (1) sporocysts with germinal masses; (2) sporocysts containing rediae; (3) immature rediae or with germinal masses; (4) rediae with daughter rediae; (5) rediae with immature cercariae; (6) rediae with mature cercariae; and (7) cercariae just shed by the snails. Nevertheless, in order to facilitate interpretation of the results, 3 categories of infection status were established according to the larval stages observed: (i) sporocysts and/or rediae with germinal masses and/or daughter rediae (stages 1–4); (ii) rediae with immature cercariae (stage 5); and (iii) rediae with mature cercariae and/or free cercariae (stages 6 and 7). There are no descriptions of the larval morphology of *C. daubneyi* available in the literature. We therefore identified the larval stages on the basis of comparison with the different stages we have previously observed in experimental infections with miracidia of this parasite (data not shown).

When sporocyst-like larval stages were observed by microscopy, samples were conserved in liquid nitrogen for subsequent molecular determination of the species using the technique developed by Martínez-Ibeas et al. [20].

### Meteorological data

Meteorological data were recorded at the weather station nearest to the farm under study, i.e. the Olas weather station, which belongs to the official meteorological service of the Galician Government [22]. The station is located at a distance of 15 km from the farm and at a similar altitude. The following meteorological variables were considered in the study: daily mean air temperature (°C), daily mean relative humidity (%), daily global solar radiation (10 kJ/m^2^day) and daily rainfall (mm). For these variables, the average or accumulated (rainfall) values corresponding to the inter-sampling periods were used. The inter-sampling periods included the days between any sampling date and the previous one. For the first sampling date, the data corresponding to the previous 30 days were considered.

### Statistical analysis

Generalized Additive Models (GAMs) [23, 24] enable assessment of associations with independent variables whose effects are not expected to remain constant along their entire range of values. We therefore used GAMs to model the association between different factors (snail size, sampling month and climatic variables) and the following response variables: (i) probability of infection (presence of *C. daubneyi* or *F. hepatica*); (ii) number of individuals collected (considered as a proxy of snail population size); and (iii) number of individuals infected with either *C. daubneyi* or *F. hepatica*.

In the probability models, we used a binomial distribution with the complementary log-log link function (clog-log), *η*_*i*_ = *log* (-*log* (*1* - (*π*_*i*_)) where *η*_*i*_ = link, and *π*_*i*_ = probability of presence, which is more appropriate for binary responses with large numbers of zeros. For the models based on counts (numbers of snails), we used a Poisson distribution with the logarithmic link. For the models of the numbers of snails infected with each parasite, we included an offset to take into account the number of snails examined on each sampling date.

In addition, we used GAMs with a gamma distribution and the identity link to explore the relationships between the sampling month and the mean number of trematode eggs per gram of faeces excreted by cows.

The smoothness selection criteria used (i.e. the rule for the degree of flexibility allowed) for the GAM models was the Unbiased Risk Estimator Criteria (UBRE) [25]. Penalised splines [26] were used as smoothers.

All analyses were implemented using the *mgcv* package [24] in R software [27]. The relationships between the same explanatory variable (e.g. sampling month) and different response variables are included in the same figure (although analyzed by different models) to enable direct comparison. For the sake of clarity, the confidence intervals are not shown. The figures corresponding to each individual model and confidence intervals are supplied as supplementary material (Additional file 1: Figures S1-S6).

## Results

### Trematode infections in intermediate hosts

A total of 2802 specimens of freshwater molluscs belonging to the species *G. truncatula* (*n* = 1141), *Radix peregra* (*n* = 1084), *Anisus* (*Anisus*) *leucostoma* (*n* = 476) and *Pisidium casertanum* (*n* = 101) were collected from the studied farm. Only the lymnaeids were found to be infected with trematode larvae. Specifically, larvae of the family Plagiorchiidae were observed in a small percentage of *R. peregra* snails (1.9%), while colonization by a greater diversity of trematodes was observed in *G. truncatula*, i.e. Notocotylidae (0.5%), Plagiorchiidae (3.4%), *F. hepatica* (4.4%) and *C. daubneyi* (8.2%). Mixed infections were never observed. Only data on *C. daubneyi* and *F. hepatica* (which are of veterinary relevance and the target of this study) and their intermediate host, *G. truncatula*, are shown.

**Fig. 1:**
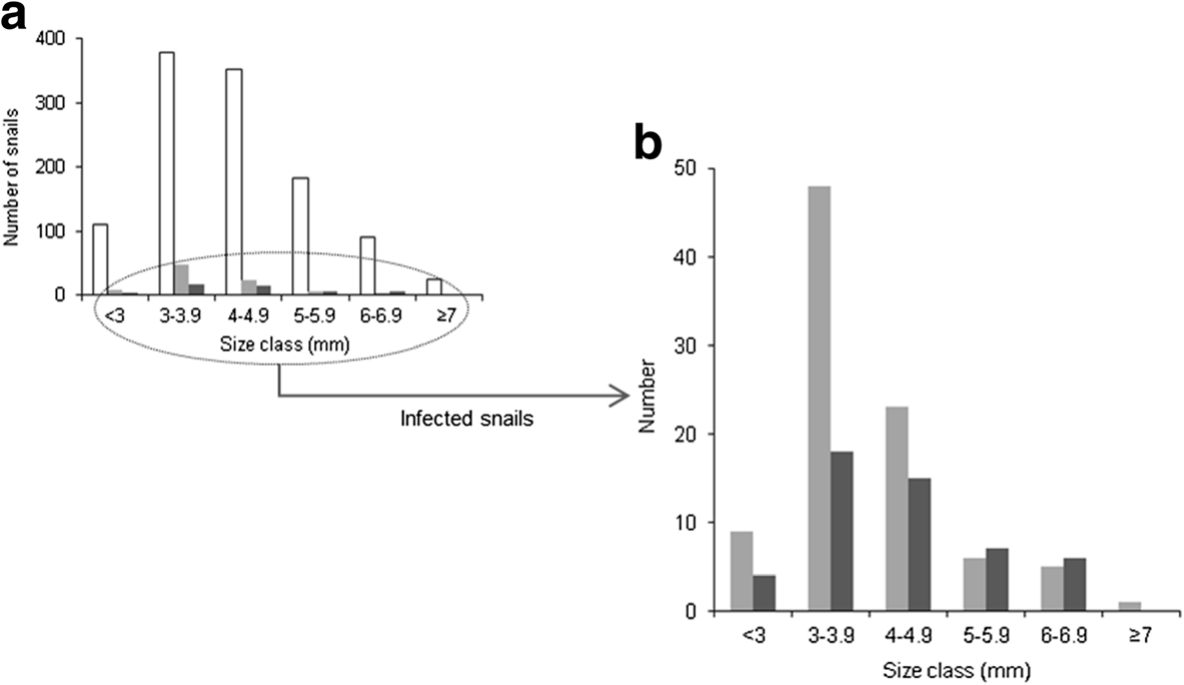
**a** Size distribution of the snail population. **b** Enlarged graph showing the number of infected individuals in each size class. Total number of snail collected (*white bar*), number of snail harbouring *C. daubneyi* (*light grey bar*), number of snails harbouring *F. hepatica* (*dark grey bar*)

To determine the effect of the snail size on the probability of infection with *C. daubneyi* and *F. hepatica*, size class distributions for the total population of snails and only infected snails were established (Fig. 1). In the total population, most individuals belonged to the 3.0–3.9 mm (33.1%) and 4.0–4.9 mm classes (30.9%) (Fig. 1a). The distribution was almost identical in snails infected with *F. hepatica*, with proportions of respectively 36.0 and 30.0% in the corresponding size classes (Fig. 1b). However, a marked skew towards the lower range was observed in the snails infected with *C. daubneyi*, so that 52.7% (49/93) were included within the 3.0–3.9 mm class.

**Fig. 2:**
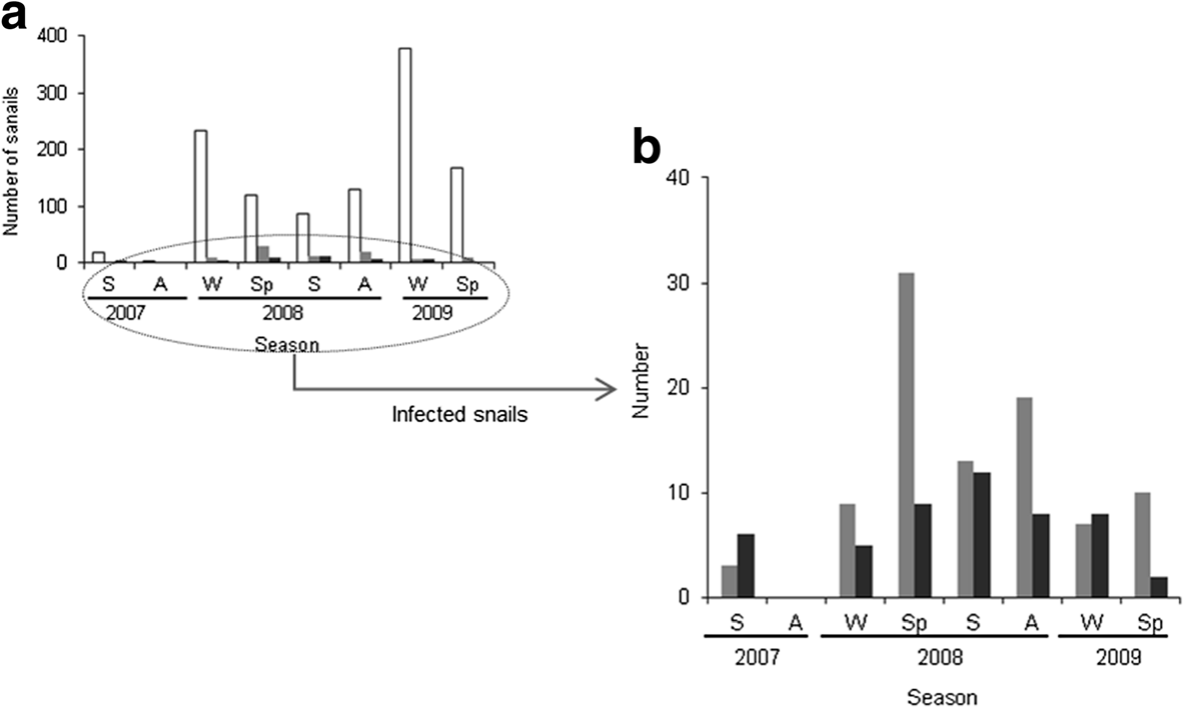
**a** Total number of *G. truncatula* collected per season. **b** Enlarged graph showing the number of parasitized snails. Total number of snails collected (*white bar*), number of snails harbouring *C. daubneyi* (*light grey bar*), number of snails harbouring *F. hepatica* (*dark grey bar*). *Abbreviations*: A, Autumn; S, Summer; Sp, Spring; W, Winter

Monthly data of *G. truncatula* abundance were grouped, for clearer presentation of results, within a seasonal framework in which winter was considered to consist of the months January, February and March, spring to comprise April, May and June, summer to include July, August and September, and autumn to comprise October, November and December. The number of snails was extremely low at the beginning of the study (summer-autumn of 2007) and then increased sharply (Fig. 2a). In all years, the total snail population increased clearly in winter, when specimens belonging to all size classes were found (Tables 1 and 2), and then decreased, with the complete disappearance of the largest snails (≥ 6 mm) during summer-autumn. A different seasonal pattern was seen when we focused only on infected snails, which were more abundant during spring-autumn of 2008, just when the total population decreased. This seasonal variation was more marked in the case of *C. daubneyi* infection (Fig. 2b).

**Table 1:** Numbers of *Galba truncatula* harbouring *Calicophoron daubneyi* according to season and snail size

		No. of snails infected/ No. of snails collected No. of snails harbouring different larval stages of the parasite						
Year	Season	< 3 mm	3–3.9 mm	4–4.9 mm	5–5.9 mm	6–6.9 mm	≥ 7 mm	Total
2007	Summer	0/1	1/9 1^c^	1/6 1^c^	1/3 1^c^			3/19 3^c^
	Autumn		0/3					0/3
2008	Winter	1/13 1^b^	0/33	4/67 2^a^ 2^b^	3/75 3^a^	1/41 1^b^	0/5	9/234 5^a^ 4^b^
	Spring	5/11 5^b^	17/39 4^a^ 13^b^	4/23 4^b^	1/12 1^b^	3/24 1^a^ 1^b^ 1^c^	1/12 1^b^	31/121 5^a^ 25^b^ 1^c^
	Summer	1/8 1^a^	10/53 7^a^ 2^b^ 1^c^	2/23 2^a^	0/3			13/87 10^a^ 2^b^ 1^c^
	Autumn	2/11 1^a^ 1^c^	13/66 3^a^ 8^b^ 2^c^	5/52 5^b^	0/2			20/131 4^a^ 13^b^ 3^c^
2009	Winter	0/46	3/105 3^a^	3/123 2^a^ 1^b^	1/75 1^c^	0/23	0/7	7/379 5^a^ 1^b^ 1^c^
	Spring	0/21	5/70 3^a^ 1^b^ 1^c^	4/58 2^a^ 1^b^ 1^c^	0/14	1/4 1^a^		10/167 6^a^ 2^b^ 2^c^
	TOTAL	9/111 2^a^6^b^1^c^	49/378 20^a^24^b^ 5^c^	23/352 8^a^ 13^b^2^c^	6/184 3^a^ 1^b^ 2^c^	5/92 2^a^ 2^b^ 1^c^	1/24 1^b^	93/1141 35^a^47^b^11^c^

^a^Sporocysts and/or rediae with germinal masses and/or daughter rediae

^b^Rediae with immature cercariae

^c^Rediae with mature cercariae and/or free cercariae

**Table 2:** Numbers of *Galba truncatula* harbouring *Fasciola hepatica* according to season and snail size

		No. of snails infected/ No. of snails collected No. of snails harbouring different larval stages of the parasite						
Year	Season	< 3 mm	3–3.9 mm	4–4.9 mm	5–5.9 mm	6–6.9 mm	≥ 7 mm	Total
2007	Summer	0/1	3/9 2^b^ 1^c^	1/6 1^b^	2/3 2^c^			6/19 3^b^ 3^c^
	Autumn		0/3					0/3
2008	Winter	1/13 1^a^	0/33	1/67 1^a^	0/75	3/41 1^b^ 2^c^	0/5	5/234 2^a^ 1^b^ 2^c^
	Spring	2/11 2^c^	3/39 1^a^ 2^b^	1/23 1^b^	2/12 2^c^	1/24 1^c^	0/12	9/121 1^a^ 3^b^ 5^c^
	Summer	0/8	7/53 6^a^ 1^b^	5/23 4^a^ 1^b^	0/3			12/87 10^a^ 2^b^
	Autumn	1/11 1^c^	3/66 1^b^ 2^c^	4/52 3^b^ 1^c^	0/2			8/131 4^b^ 4^c^
2009	Winter	0/43	2/105 1^b^ 1^c^	2/123 1^a^ 1^c^	2/75 1^b^ 1^c^	2/23 1^a^ 1^c^	0/7	8/379 2^a^ 2^b^ 4^c^
	Spring	0/21	0/70	1/58 1^c^	1/14 1^b^	0/4		2/167 1^b^ 1^c^
	Total	4/111 1^a^3^c^	18/378 7^a^ 7^b^ 4^c^	15/352 6^a^ 6^b^ 3^c^	7/184 2^b^ 5^c^	6/92 1^a^ 1^b^ 4^c^	0/24	50/1141 15^a^16^b^ 19^c^

^a^Sporocysts and/or rediae with germinal masses and/or daughter rediae

^b^Rediae with immature cercariae

^c^Rediae with mature cercariae and/or free cercariae

**Fig. 3:**
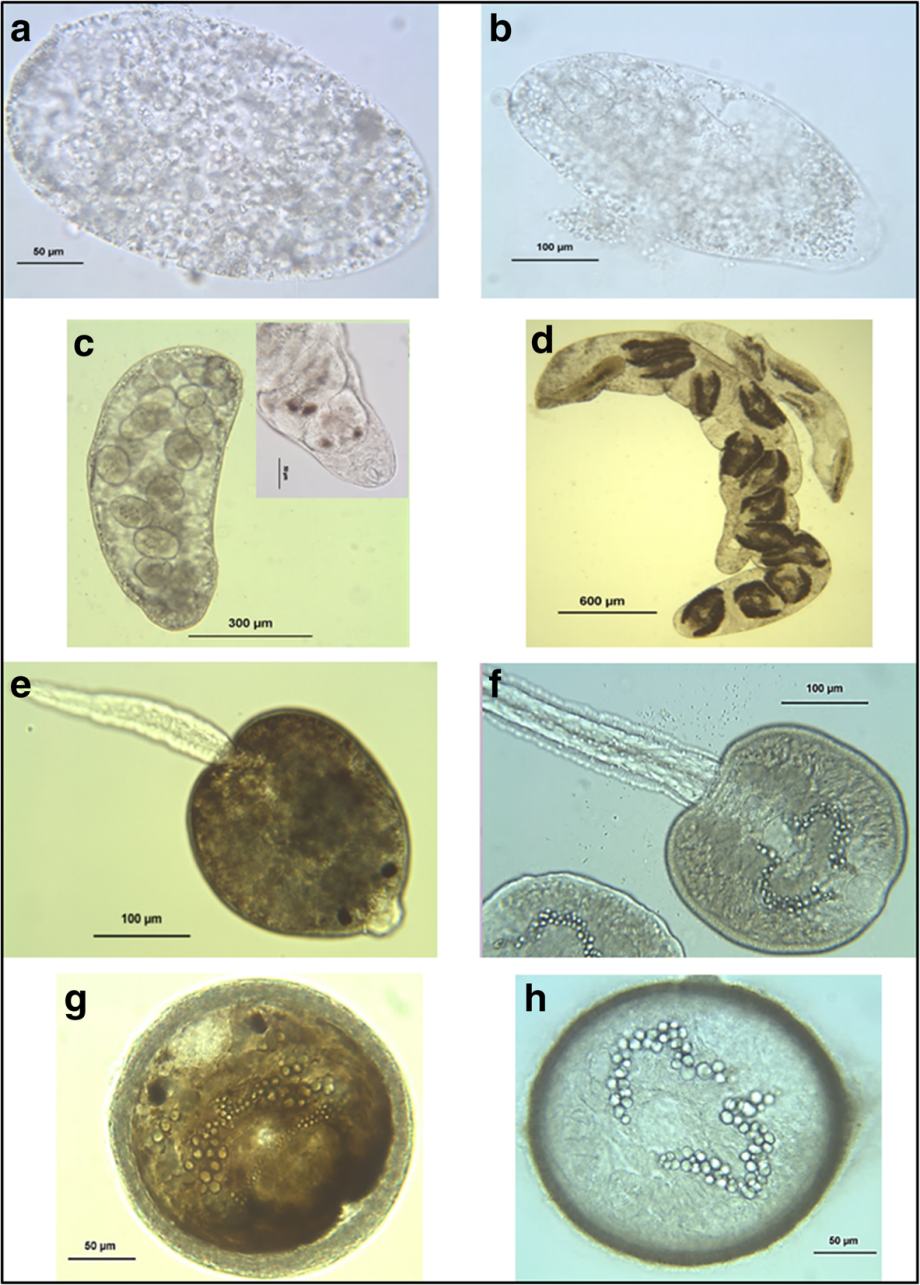
Larval stages of *C. daubneyi* and *F. hepatica*. **a** Sporocyst of *C. daubneyi*. **b** Sporocyst of *F. hepatica* containing redia. **c** Redia of *C. daubneyi* with detail showing the anterior portion (insert). **d** Rediae of *F. hepatica* (different generations within the same snail). **e** Mature cercaria of *C. daubneyi*. **f** Mature cercaria of *F. hepatica*. **g** Metacercaria of *C. daubneyi.* **h** Metacercaria of *F. hepatica*. Larval stages in **a** and **b** were confirmed by molecular techniques according to Martínez-Ibeas et al. [20]. Larval stages in **c**-**h** were classified morphologically by microscopic techniques. Mature cercariae released during dissection of infected snails encysted on the slides to form the metacercariae shown in **g**-**h**. All photographs are original work by Yolanda Manga-González

Detailed microscopic examination of the infected snails enabled us to determine the different stages of larval developmental (see Methods and Fig. 3). Tables 1 and 2 summarize the infection status of the snails in relation to their size and sampling season. All size classes of snails, except the > 7 mm class, harboured larval stages ranging from barely evolved (indicative of recent or arrested infections) to fully developed. Among the different size classes, the proportion of infected snails harbouring mature cercariae varied widely [ranges for *C. daubneyi*: 8.7% (2/23) – 33.3% (2/6); ranges for *F. hepatica*: 20.0% (3/15) – 75.0% (3/4)]. However, the absolute number of snails with mature cercariae within each class was similar (ranges: 1–5 for *C. daubneyi*; 3–5 for *F. hepatica*). All size classes should have a similar capacity to contaminate pastures. Considering the season, some differences were observed between both parasites, especially after the winter of 2008 when the snail population increased significantly. For *C. daubneyi* (Table 1), the three categories of infection status appeared continuously from spring 2008, while for *F. hepatica* (Table 2), some developmental stages were absent in some seasons. Specifically, mature cercariae were not found in the summer of 2008, and barely evolved larval stages were not detected in the autumn of 2008 or spring of 2009.

### Patency of *C. daubneyi* and *F. hepatica* infections in definitive hosts

**Fig. 4:**
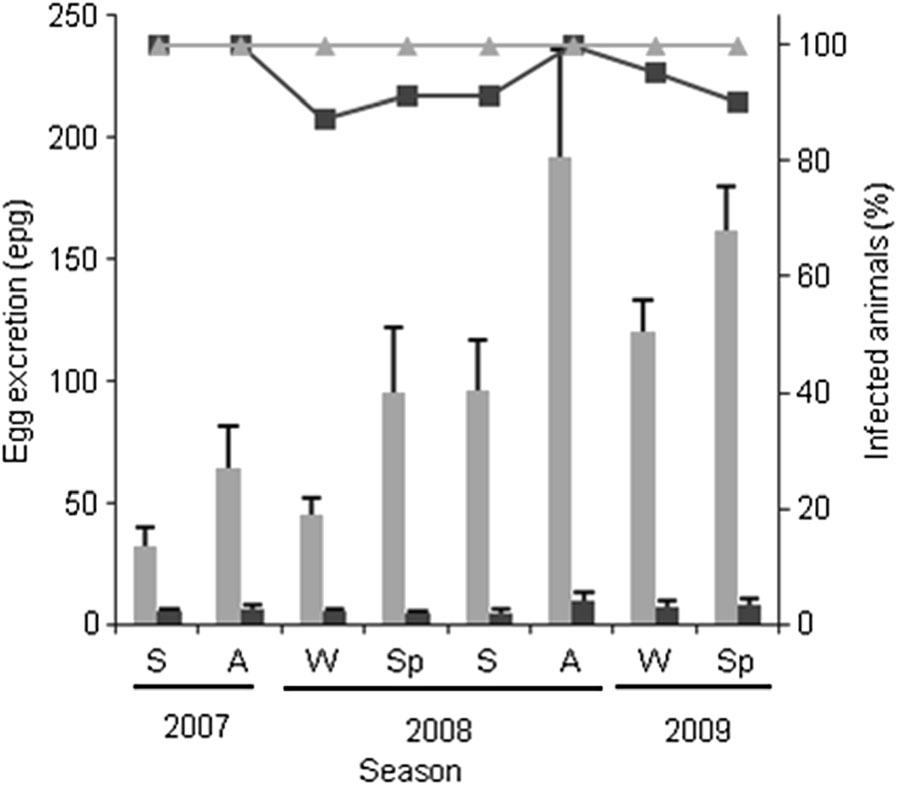
Seasonal variation in excretion of *C. daubneyi* (*light grey*) and *F. hepatica* (*dark grey*) eggs in cow faeces. Percentage of positive cows (lines) and mean counts ± standard deviation, SD (bars). *Abbreviations*: A, Autumn; S, Summer; Sp, Spring; W, Winter

The seasonal variation in excretion of eggs of both flukes *via* cow faeces, observed during the study, is shown in Fig. 4. All cows were positive for *C. daubneyi* eggs in all seasons, while some variations were observed in the patency of *F. hepatica* infection, probably because the number of eggs was close to the detection limit of the coprological technique on some sampling occasions. The *F. hepatica* egg counts were always very low, with mean values ranging from 4.2 ± 1.3 (± standard deviation, SD) epg, in spring 2008, to 9.5 ± 3.8 epg, in autumn of the same year. The *C. daubneyi* egg counts were higher, with mean values ranging from 32.6 ± 7.8, in summer 2007, to 191.9 ± 44.2, in autumn 2008. Mixed infections by both flukes were detected in all animals in summer-autumn 2007 and in autumn 2008.

### Modelling *C. daubneyi* and *F. hepatica* infections in intermediate and definitive hosts

**Fig. 5:**
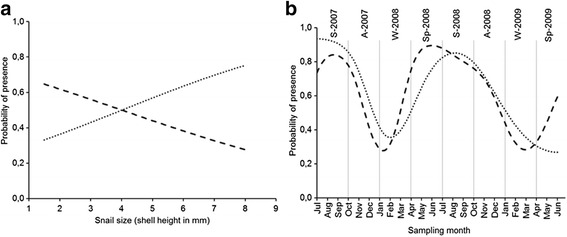
Relationship between the probability of infection of *G. truncatula* by fluke larvae and snail size (**a**) and sampling month (**b**) estimated through the smooth effect(*s*) yielded by the GAMs shown in Table 3. The dashed line represents *C. daubneyi* data and the dotted line, *F. hepatica* data. The seasons, separated by vertical lines, are indicated at the top of graph **b**. *Abbreviations*: A, Autumn; S, Summer; Sp, Spring; W, Winter

The results of GAMs for the probability of infection of *G. truncatula* in relation to the snail size and collection month are shown in Table 3. The probability of infection with *C. daubneyi* larvae was significantly associated with both variables, while the effect of the size was only marginally significant (*P* = 0.058) for infection with *F. hepatica*. The presence of larvae of both trematode species in the snails was linearly related to snail size, but with opposing trends (Fig. 5a). Thus, the probability of infection with *C. daubneyi* decreased and the probability of infection with *F. hepatica* increased as snail size increased. Regarding the temporal variation in the probability of infection over the study period, similar patterns were observed for both trematodes (Fig. 5b). Thus, the probability of infection increased during spring-summer, reaching values above 0.8, and then decreased in autumn to minimal values of < 0.4 in winter. In both models, the percentage of explained deviance was 19.0% for *C. daubneyi* and 12.8% for *F. hepatica*.

**Table 3:** Estimated components of the GAMs for the relationships between the probability of infection by trematode larvae and snail size and sampling month, with corresponding effective degrees of freedom (edf), Chi-square statistic, *P-*values, and percentage of deviance explained by the models (%DE)

Smoothing effect(*s*) variable	Probability of infection							
	*C. daubneyi*				*F. hepatica*			
	*edf*	*χ* ^2^	*P*	%DE	*edf*	*χ* ^2^	*P*	%DE
Snail size	1.0	4.6	0.035	19.0	1.0	3.6	0.058	12.8
Sampling month	6.8	92.8	< 0.001		5.4	49.9	< 0.001

The seasonal changes in the snail population will determine the period during which trematode life-cycles can continue. This will, in turn, determine the seasonal abundance of infected snails and ultimately the risk of infection of the definitive host throughout the year. GAM analysis was also used to model the abundance of snails (total population and number of specimens infected with *C. daubneyi* and *F. hepatica*) in relation to time of year. The sampling month proved to be a highly significant explanatory variable for snail abundance, accounting for a high percentage (71.9–78.2%) of the observed variability in the three populations studied (Table 4).

**Table 4:** Estimated components of the GAMs for the relationships between snail abundance and sampling month, with corresponding effective degrees of freedom (edf), Chi-square statistic, *P-*values, and percentage of deviance explained by the models (%DE)

Smoothing effect (*s*) variable	Number of snails											
	Total no. collected				No. harbouring *C. daubneyi*				No. harbouring *F. hepatica*			
	*edf*	*χ* ^2^	*P*	%DE	*edf*	*χ* ^2^	*P*	%DE	*edf*	*χ* ^2^	*P*	%DE
Sampling month	8.9	438.9	< 0.001	78.2	6.7	76.1	< 0.001	71.9	4.1	37.5	< 0.001	72.2

**Fig. 6:**
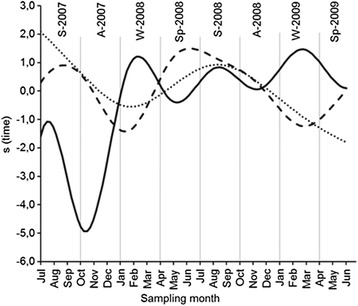
Temporal variation in the snail populations according to the estimated centred smooth effect(*s*) yielded by the GAMs shown in Table 4. The continuous line represents the total number of snails, the dashed line represents the number of snails harbouring *C. daubneyi* and the dotted line, the number of snails harbouring *F. hepatica*. Seasons, separated by vertical lines, are indicated at the top of the figure. *Abbreviations*: A, Autumn; S, Summer; Sp, Spring; W, Winter

**Fig. 7:**
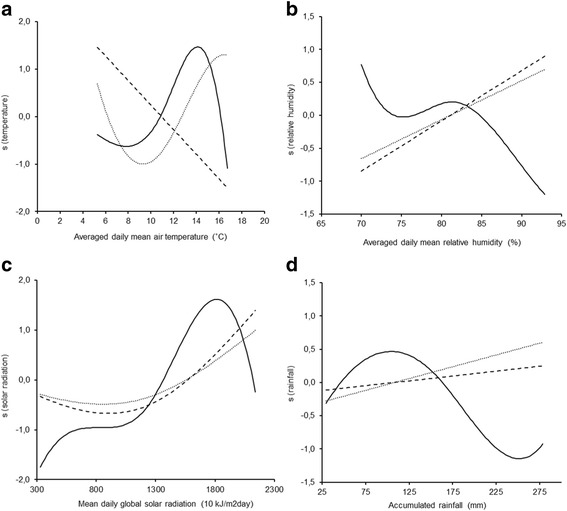
Relationships between snail abundance and climatic variables according to the estimated centred smooth effect (*s*) obtained by the GAMs shown in Table 5. **a** Averaged daily mean air temperature. **b** Averaged daily mean relative humidity. **c** Mean daily global solar radiation. **d** Accumulated rainfall. The continuous line represents the total number of snails, the dashed line represents the number of snails harbouring *C. daubneyi*, and the dotted line, the number of snails harbouring *F. hepatica*

The observed seasonality in the abundance of snails (Fig. 6) led us to explore the effect of the available climatic variables, by including them in the models (which originally only included time as an independent variable). Because of concurvity in the climatic data, each climatic variable was included separately in the models. The total snail numbers were significantly related to all climatic variables, and the abundance of snails infected with each of the trematode species was also significantly associated with all climatic variables, except accumulated rainfall (Table 5). Moreover, the inclusion of the climatic variables in the GAMs increased the percentage of explained deviance in all models, except in the GAMS for the number of *C. daubneyi*-infected snails, which included the mean daily global solar radiation and the accumulated rainfall. Considering the percentage of explained deviance as a fitting criterion, the best fits were provided by the following models: (i) for the total population of snails, the model including the global solar radiation (explained deviance = 91.9%; model 3); (ii) for the number of *C. daubneyi*-infected snails, the model including mean temperature (explained deviance = 77.7%; model 1); and (iii) for the number of *F. hepatica*-infected snails, the model fitted with the mean relative humidity (explained deviance = 83.2%; model 2). The individual effects of the climatic variables on the snail populations are shown in Fig. 7. Those associated with temperature, relative humidity and solar radiation deserve special attention due to the observed significant effects on the three snail populations studied. The number of snails infected with *C. daubneyi* was negatively and linearly related to temperature. By contrast, the number of snails infected with *F. hepatica* and the total snail population were positively related to temperatures ranging from 10–16 °C and from 8–14 °C, respectively (Fig. 7a). The number of snails infected with each trematode species was positively associated with the relative humidity, whereas the total population was negatively related to relative humidity, mainly at values exceeding 80% (Fig. 7b). The size of the three snail populations was positively related to solar radiation, although the effect on the infected populations was weaker (Fig. 7c).

**Table 5:** Estimated components of the GAMs for the relationships between snail abundance and climatic variables and time, with corresponding effective degrees of freedom (edf), Chi-square statistic, *P-*values, and percentage of deviance explained by the models (%DE)

Smoothing effect (*s*) variable	Number of snails											
	Total no. collected				No. harbouring *C. daubneyi*				No. harbouring *F. hepatica*			
	*edf*	*χ* ^2^	*P*	%DE	*edf*	*χ* ^2^	*P*	%DE	*edf*	*χ* ^2^	*P*	%DE
Model 1												
Mean temperature	3.88	91.7	< 0.001	89.1	1.0	4.9	< 0.05	77.7	2.9	33.6	< 0.001	79.2
Sampling month	8.82	367.5	< 0.001		6.77	47.2	< 0.001		1.0	8.18	< 0.01	
Model 2												
Mean relative humidity	3.7	45.7	< 0.001	83.5	1.0	7.2	< 0.01	76.0	1.0	3.3	< 0.01	83.2
Sampling month	8.8	291.8	< 0.001		6.2	68.8	< 0.001		5.2	37.8	< 0.001	
Model 3												
Global solar radiation	3.9	88.0	< 0.001	91.9	1.9	13.3	< 0.01	71.6	1.7	5.5	0.049	76.8
Sampling month	8.9	331.4	< 0.001		4.1	33.9	< 0.001		3.2	22. 8	< 0.001	
Model 4												
Accumulated rainfall	3.7	45.9	< 0.001	84.0	1.0	0.3	0.580	71.9	1.0	1.5	0.226	80.4
Sampling month	8.8	370.5	< 0.001		6.6	65.4	< 0.001		5.1	35.4	< 0.001

**Fig. 8:**
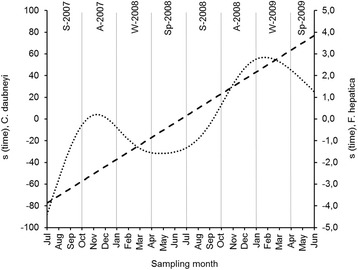
Temporal variation in the mean epg (eggs per gram of faeces) according to the estimated centred smooth effect(*s*) yielded by the GAMs. The dashed line represents the *C. daubneyi*-epg, and the dotted line, the *F. hepatica*-epg. Seasons, separated by vertical lines, are indicated at the top of the graph. *Abbreviations*: A, Autumn; S, Summer; Sp, Spring; W, Winter

In the definitive hosts, the mean number of fluke eggs in the faeces was significantly associated with the sampling month. The effect was more marked for *C. daubneyi* (*edf* = 1.0; Ref. *df* = 1.0; *F* = 34.78; *P* < 0.001; explained deviance = 57.1%) than for *F. hepatica* (*edf* = 4.89; Ref. *df* = 5.94; *F* = 5.29; *P* < 0.01; explained deviance = 56.8%). For both trematodes, egg excretion tended to increase throughout the study period (Fig. 8). Nevertheless, the relationship (linear) was much more constant and stronger for *C. daubneyi* than for *F. hepatica* (see shape of curves and scale of y axes).

## Discussion

Changes in the populations of freshwater molluscs and the natural infections associated with *C. daubneyi* and *F. hepatica* were studied throughout 2 years on a cattle farm where both infections coexisted. As both parasites and molluscs shared the habitat and were thus exposed to the same environmental conditions (changes in weather, physicochemical variables and/or biocoenosis), any difference in the dynamics of these infections could be attributed to particular behavioural traits of the different molluscan and trematode species involved. Although all species of molluscs sampled were exposed to the same risk of infection by miracidia, *G. truncatula* was the only species involved in the transmission of *C. daubneyi* and *F. hepatica*. This reveals the importance of this snail as an intermediate host for *F. hepatica*, as is well known, and also for *C. daubneyi*, a parasite whose epidemiology has scarcely been studied until now. In addition, we analyzed 1097 freshwater gastropods belonging to the species *R. peregra*, *P. casertanum*, *Potamopyrgus antipodarium*, *Anisus anisus*, *Physa fontinalis* and *Succinea putris*, collected from other farms with cattle infected by *C. daubneyi* and *F. hepatica*. We did not find larval stages of either parasite in any of the specimens. The involvement of species other than *G. truncatula* in the transmission of both trematodes in this region can therefore be ruled out. In the UK, Jones et al. [18] also found that *C. daubneyi* infected *G. truncatula* but not *R. balthica* or *P. antipodarum* collected from the same habitats. Another lymnaeid snail, *Lymnaea glabra*, has also been reported to be a natural host for *C. daubneyi* and *F. hepatica* in France [21, 28]. Nevertheless, Abrous et al. [15] showed that the role of this snail species as a transmitter was very limited (prevalence very low or nil) when *G. truncatula* was also present in the same habitat. Our findings in relation to *F. hepatica* do not appear to be consistent with those of Relf et al. [17] and Jones et al. [18], who detected parasite DNA in a high proportion of *R. peregra* snails in Ireland (66 out of 167) and the UK (13 out of 52). Nevertheless, the presence of live *F. hepatica* larvae in these snails was not definitely demonstrated (i.e. by microscopic visualization), and the DNA-positive reactions may reflect unsuccessful infections in which the miracidia entered the snail but were not able to develop at all or only did so up to very early larval stages. Indeed, sporocyst encapsulation has frequently been observed in *R. peregra* snails experimentally exposed to the miracidia of *F. hepatica* [29]. Such microscopically undetectable, abortive early infections cannot be ruled out in the present study. However, we believe that the examination of such a large number of *R. peregra* specimens allows us to state that this snail species does not transmit *F. hepatica* in Galicia.

The rates of infection with *C. daubneyi* and *F. hepatica* were much lower in the intermediate host (8.1 and 4.4%, respectively) than in the definitive host (all cows shed eggs of both parasites throughout the study period). According to the published data, similar or even lower prevalence rates are generally observed for infections caused by a great variety of trematode species, including *C. daubneyi* and *F. hepatica*, in lymnaeid populations in natural ecosystems [14, 16, 18, 30]. In a retrospective study carried out in Central France over a 12-year period, differences in the prevalence of *C. daubneyi* infection were observed in the definitive and intermediate hosts. Thus, the prevalence increased steadily in cattle (from 5.23 to 44.72%), while it fluctuated between low values in the snails (between 0.8 and 6.3%) [31]. This may be attributed to different factors depending on snails and parasites and their complex relationships. For instance, in some snail-trematode systems, it has been demonstrated experimentally that some non-host snail species (resistant to infection) can act as a decoy, thus interfering and consequently reducing the rate of infection of the host species with which the habitat is shared [32]. Populations with phenotypic differences in susceptibility to infection by certain trematode species have also been described within the same species [33]. The susceptibility has been shown to be associated with expression of certain stress genes, in the case of the snail *Biomphalaria glabrata* and the parasite trematode *Schistosoma mansoni* [34, 35]. Finally, the findings of the present study confirm the rarity of natural co-infections with different digenean species [20, 21, 36], probably as a result of competition, which would play an important role in the configuration of trematode infection status of snail populations in each habitat. The mechanisms underlying competitive processes remain largely unknown; however, such mechanisms may constitute a basis for developing strategies to interrupt the life-cycle of trematodes. Further research is needed to provide data on which to base improvements in the control of paramphistomosis and fasciolosis, against which no vaccines or effective treatments are currently available.

The dynamics of natural infections by *C. daubneyi* and *F. hepatica* in their intermediate snail hosts were also addressed with the ultimate aim of constructing statistical models to estimate the risk of transmission to cattle in the area. Specifically, two infection variables were followed-up over time: the probability that an individual is infected and the number of individuals infected within the whole population. For the probability of infection, associations were observed with both snail size and sampling month. However, some differences between the two parasites were observed, mainly in relation to snail size, which was positively associated with the probability of presence of *F. hepatica*, but negatively associated with that of *C. daubneyi*. The findings in relation to *F. hepatica* infection seem logical as the larger (i.e. older) snails may have been exposed for longer to miracidia, with a consequently higher probability of infection [14]. Moreover, the larger size of the host may provide more resources in terms of space and/or nutrients for the growth and reproduction of the parasite larvae [37–39]. In this regard, castration of the host snails by trematodes, which seems to be a common phenomenon, causes gigantism and thus enables larger parasite burdens [40–42]. The observations in relation to *C. daubneyi* may be the consequence of the development, by the snails, of age-dependent resistance to the miracidial infection and/or the result of the stunted growth induced by the parasite larvae in susceptible snails. By means of experimental infections, Dar et al. [43] demonstrated the existence of age-related resistance to *C. daubneyi* infection in the lymnaeid *Pseudosuccinea columella*: only the smallest snails of shell height ≤ 2 mm at miracidial exposure were able to sustain complete larval development of the parasite until cercarial shedding. Indeed, Rondelaud et al. [19] observed that *C. daubneyi* caused more severe kidney damage than *F. hepatica*, in experimentally-infected snails. The behavioural differences in both parasites within *G. truncatula* may be explained in terms of host/parasite adaptation, and better adaptation would have been reached by *F. hepatica* in the host, at least in the study area.

The association between probability of infection in snails and sampling month was very strong, with similar seasonal variations for both parasites (high probability of infection in spring-summer). Nonetheless, in 2008 the probability of infection by *C. daubneyi* increased earlier (spring) than the probability of infection by *F. hepatica* (summer). Judging by the changes in the snail population, it appears that many eggs hatched during the winter, giving rise to a new snail population susceptible to miracidial infection. Although eggs of both trematodes were regularly excreted by cattle, *C. daubneyi* eggs were always the most abundant, and the probability of infection by this species would therefore expected to be higher.

The temporal variability in the populations of intermediate hosts in the habitat will determine the magnitude of the population susceptible to parasitization and, ultimately, the risk of transmission to the definitive host. The life-cycle of *G. truncatula* showed a seasonal pattern in the study area, and the reproduction and growth rates were highest in winter-spring. Two factors were noted during this period: an increase in the total population and the presence of snails of all size classes. The population increase that occurred in the winter of 2008 was particularly notable, as the population had been close to extinction in the previous months (summer-autumn). In *G. truncatula* habitats, recolonization after drastic reductions in population size is common, due to the self-fertilizing ability of this snail species [44–46]. The changes in the snail numbers observed in the present study indicate that the prevalence of infection alone (i.e. without taking into account the fluctuations in the snail density in the habitat) is not sufficient to assess the risk of transmission to the definitive host. Although this parameter is traditionally used in epidemiological studies of intermediate hosts, it should be replaced by another parameter such as the absolute abundance of infected snails, which provides a more realistic measure of the risk of infection in cattle.

The number of infected snails, particularly those infected with *C. daubneyi*, also underwent seasonal fluctuations. Specifically, the numbers increased significantly in spring-autumn, probably because many snails were infected in winter-spring, when the total population was larger. Transmission of both parasites to the definitive host could occur at any time, as snails harbouring rediae with mature cercariae or free cercariae ready to be released to the environment (where they encyst and persist for long periods as infective metacercariae) were found in all seasons. This is consistent with the pattern of egg excretion observed in the definitive host. Importantly, in the case of *C. daubneyi*, the faecal egg counts were closely correlated with the number of parasites in the rumen [8, 13, 47]. A gradual increase in egg shedding would reflect a cumulative increase in fluke burdens due to continuous reinfection. This cannot be inferred for *F. hepatica* because the egg counts are not correlated with worm burdens in the liver [48, 49].

The associations between climate factors and trematode transmission dynamics are generally accepted and have been studied for quite some time [50]. However, the statistical techniques traditionally used to assess these associations, such as linear regression models, are limited in their ability to fit the often complex non-linear relationships between these variables and the host snail-trematode interaction, a key link in the transmission chain. More realistic descriptions of these phenomena in natural ecosystems can be obtained by using GAM models, as done in the present study.

The variations in snail populations were significantly associated with four meteorological variables, although these only accounted for a low percentage (maximum of about 13%) of the seasonal deviance explained by the models. The total snail population increased with increasing temperature and solar radiation up to 14 °C and 18,000 KJ respectively; this effect was expected, given the positive influence of both variables on the growth of microalgae on which snails feed, and their positive effect on activity, feeding and growth of the ectothermic intermediate host [51]. However, the opposite effect was observed in relation to higher temperature and solar radiation. According to the historical climatic records available for the study area (10 years; Additional file 1: Figure S7), such high average values only occur between July and September, during which the lowest rainfalls are also recorded. In addition to the high permeability of most soils in the region, this leads to rapid draining and desiccation of the habitats, making them unsuitable for snails, of which only low numbers survive by aestivation. Considering the accumulated rainfall, a bimodal effect was also observed, as the total snail numbers increased with increasing rainfall, until reaching a maximum at approximately 100 mm per month; however, the numbers decreased when monthly precipitation exceeded 125 mm, probably because many snails were washed away by the heavy rains. A negative effect was also observed for the relative air humidity (over 80%) associated with periods of high rainfall.

Some associations were observed between abundance of infected snails and climatic factors. The effect of temperature was especially striking, as contrasting responses were observed for each infection. Thus, the snail population infected with *F. hepatica* increased, whilst the snail population infected with *C. daubneyi* decreased as the temperature increased. The behaviour of snails infected with *F. hepatica* was consistent with that observed in this study for the total population and with previous reports on the development of experimental infections in snails maintained within the same temperature range [52, 53]. The unusual behaviour of population infected with *C. daubneyi* may be related to the needs of the free-living stages and/or the suitability of *G. truncatula* as a host for this parasite. It has not been demonstrated that *C. daubneyi* requires lower temperatures than *F. hepatica* for the development and hatching of the eggs [54]. Moreover, the combined action of the aforementioned phenomena (greater susceptibility of younger snails to infection and stunted growth caused by the parasite larvae) may explain the negative association between the temperature and the abundance of snails infected with *C. daubneyi*. According to the phenological cycle of *G. truncatula* in this habitat, the snail hatching rate is highest in winter, so that larger numbers of young specimens susceptible to being infected would be present in the coldest periods. Regarding variables related to the water availability in the environment, i.e. relative humidity and the accumulated rainfall, the positive influence on populations of snails infected with both parasites seems to confirm that the negative effects on the total snail population were due to the heavy rain. Snails that were not washed away found damp habitats that proved suitable for maintaining infections [55].

## Conclusions

To our knowledge, this is the first exhaustive study on the temporal follow-up of infections caused by *C. daubneyi* and *F. hepatica* in molluscs and cattle in a natural setting. Moreover, the seasonal variations in snail populations, including those infected with both trematodes were statistically modelled for the first time in Galicia, a region where paramphistomosis and fasciolosis are endemic in cattle. This enabled us to identify some risk periods that should be taken into account when designing control programmes. Thus, special care should be taken to prevent pastures and the surrounding environment being contaminated with parasite eggs during winter-spring, when the numbers of snails susceptible to miracidial infections are maximal. This time of year is therefore the optimal moment for treating grazing animals. Nevertheless, control of trematodosis based only on chemotherapy is difficult in an area such as this where the environmental factors favour the regular appearance of snail populations harbouring completely developed larval stages, which would contaminate the pasture. Novel preventive strategies must therefore be designed, including biological control and identification of resistant genotypes of snails for introduction into natural systems.

## Additional file

Additional file 1: Figure S1. Probability of infection with the trematodes *C. daubneyi* (**a**, **b**) and *F. hepatica* (**c**, **d**) in the snail *G. truncatula*, in relation to snail size (**a**, **c**) and sampling month (**b**, **d**), estimated by the smoothing effect(s) in GAMs. **Figure S2.** Temporal variation in snail populations according to the centred smoothing effect(s) in the GAMs: total number (**a**), numbers harbouring *C. daubneyi* (**b**) and *F. hepatica* (**c**). **Figure S3.** Relationships between snail abundance and averaged daily mean air temperature (**a**), averaged daily mean relative humidity (**b**), mean daily global solar radiation (**c**) and accumulated rainfall (**d**). **Figure S4.** Relationships between the abundance of snails infected with *C. daubneyi* and averaged daily mean air temperature (**a**), averaged daily mean relative humidity (**b**), mean daily global solar radiation (**c**) and accumulated rainfall (**d**). **Figure S5.** Relationship between the abundance of snails infected with *F. hepatica* and averaged daily mean air temperature (**a**), averaged daily mean relative humidity (**b**), mean daily global solar radiation (**c**) and accumulated rainfall (**d**). **Figure S6.** Temporal variation in the mean number of eggs per gram of faeces (epg) according to the centred smooth effect(s) estimated by the GAMs. **Figure S7.** Monthly averaged values of daily mean air temperature (**a**) and daily global solar radiation (**b**), and monthly accumulated rainfall (**c**) recorded in the study area (Olas weather station) for the period July 2006 to June 2016. (PDF 1987 kb)

## Abbreviations

epg: Eggs per gram of faeces
GAM: Generalized additive models
IH: Intermediate host
UBRE: Unbiased risk estimator criteria
