# Supplementary material for: Transmission of Calicophoron daubneyi and Fasciola hepatica in Galicia (Spain): Temporal follow-up in the intermediate and definitive hosts
Source: Parasit Vectors. 2016 Nov 29;9:610. doi: 10.1186/s13071-016-1892-8 (PMC5126829; doi:10.1186/s13071-016-1892-8)

Supporting Information

Figure S1 Probability of infection with the trematodes *C. daubneyi* (a, b) and *F. hepatica* (c, d) in the snail *G. truncatula*, in relation to snail size (a, c) and sampling month (b, d), estimated by the smoothing effect (s) in GAMs. Shading indicates the 95% confidence bands. Vertical lines in c and d separate seasons in the study period.

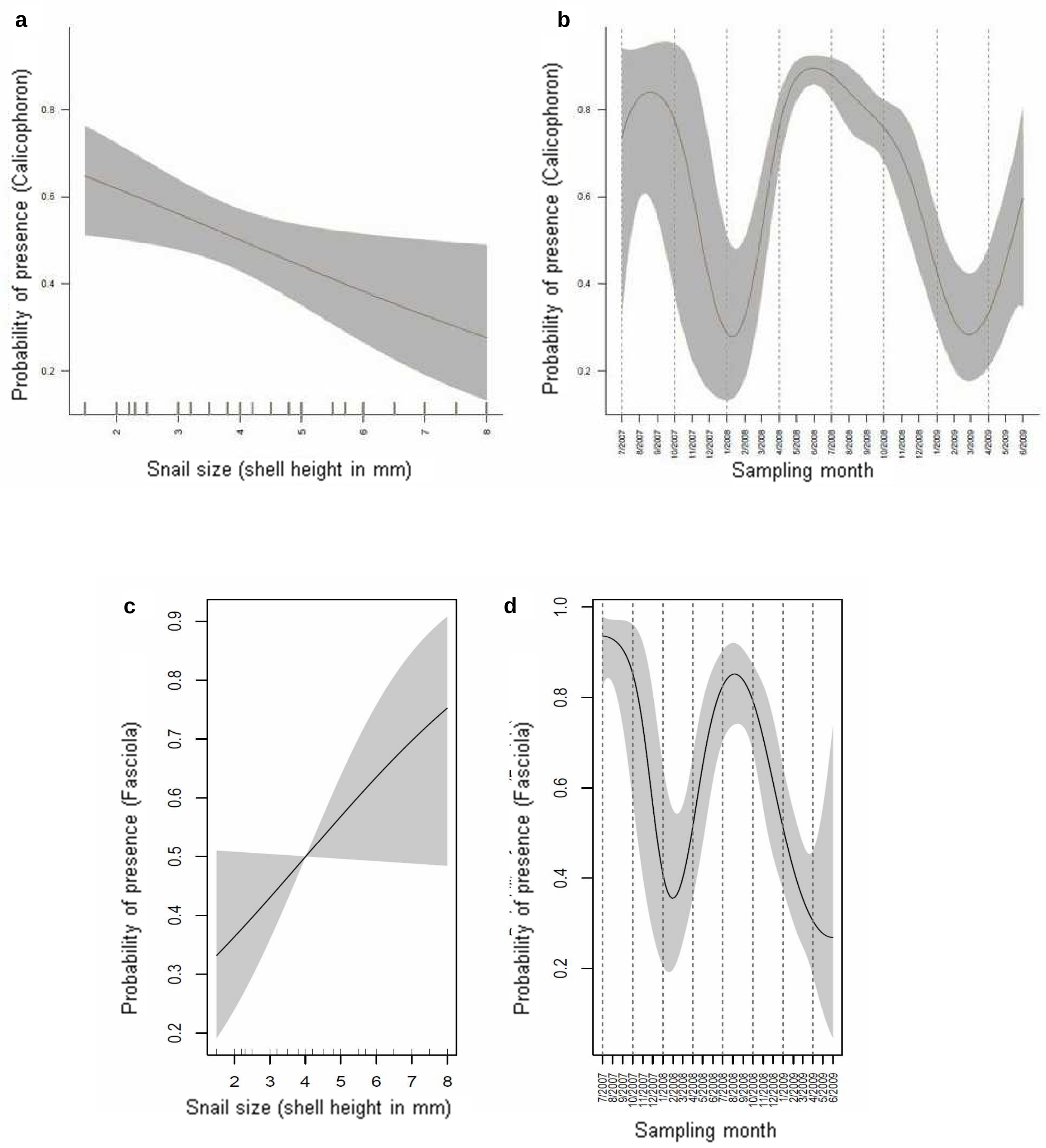

Figure S2 Temporal variation in snail populations according to the centred smoothing effect (s) in the GAMs: total number (a), numbers harbouring *C. daubneyi* (b) and *F. hepatica* (c). Numbers on the *y* axis indicate the effective degrees of freedom (edf) for each effect. Shading indicates the 95% confidence bands. Vertical lines separate seasons considered in the study period.

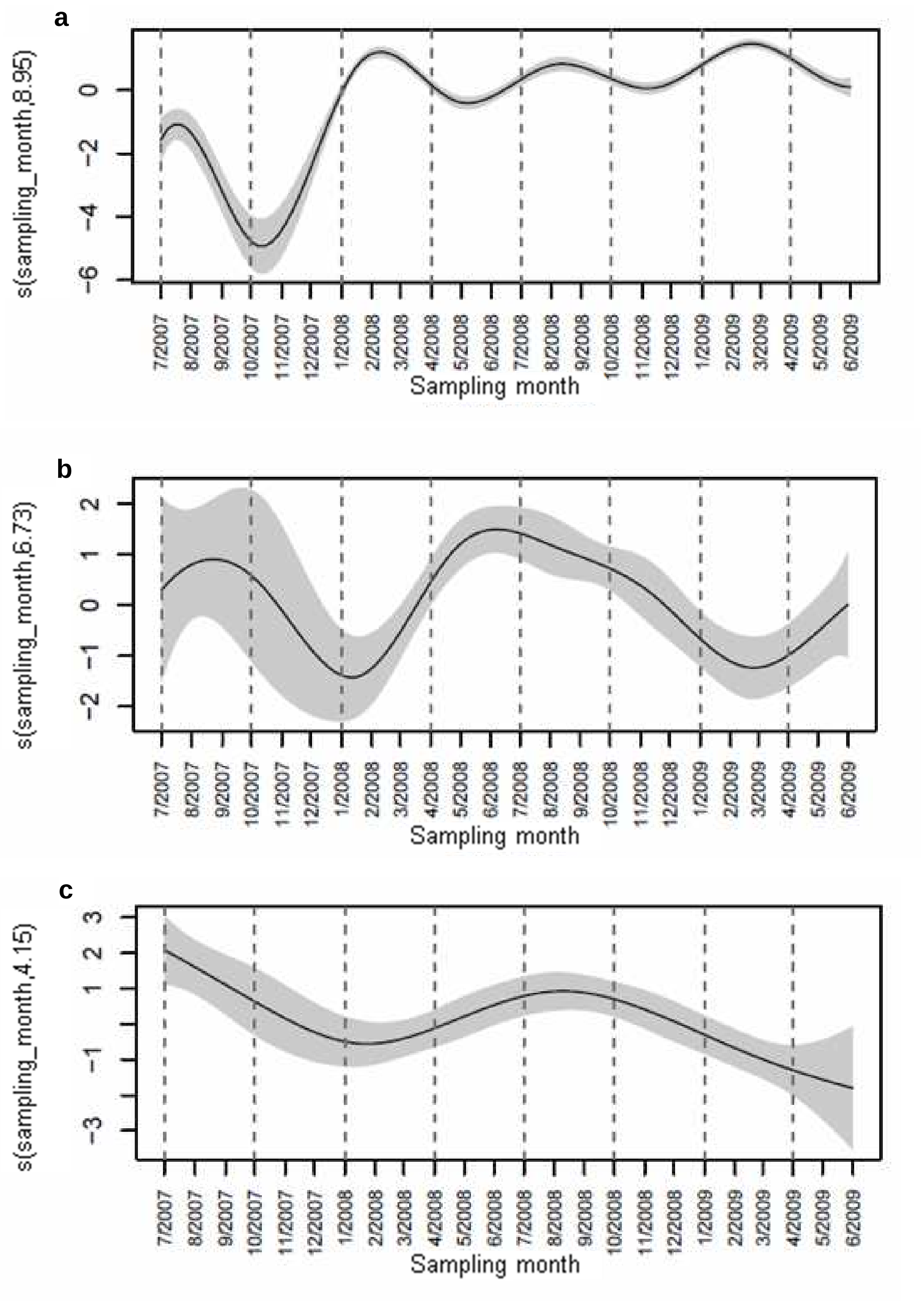

Figure S3 Relationships between snail abundance and averaged daily mean air temperature (a), averaged daily mean relative humidity (b), mean daily global solar radiation (c) and accumulated rainfall (d). Numbers on the *y* axis indicate the effective degrees of freedom (edf) for each effect. Shading indicates the 95% confidence bands.

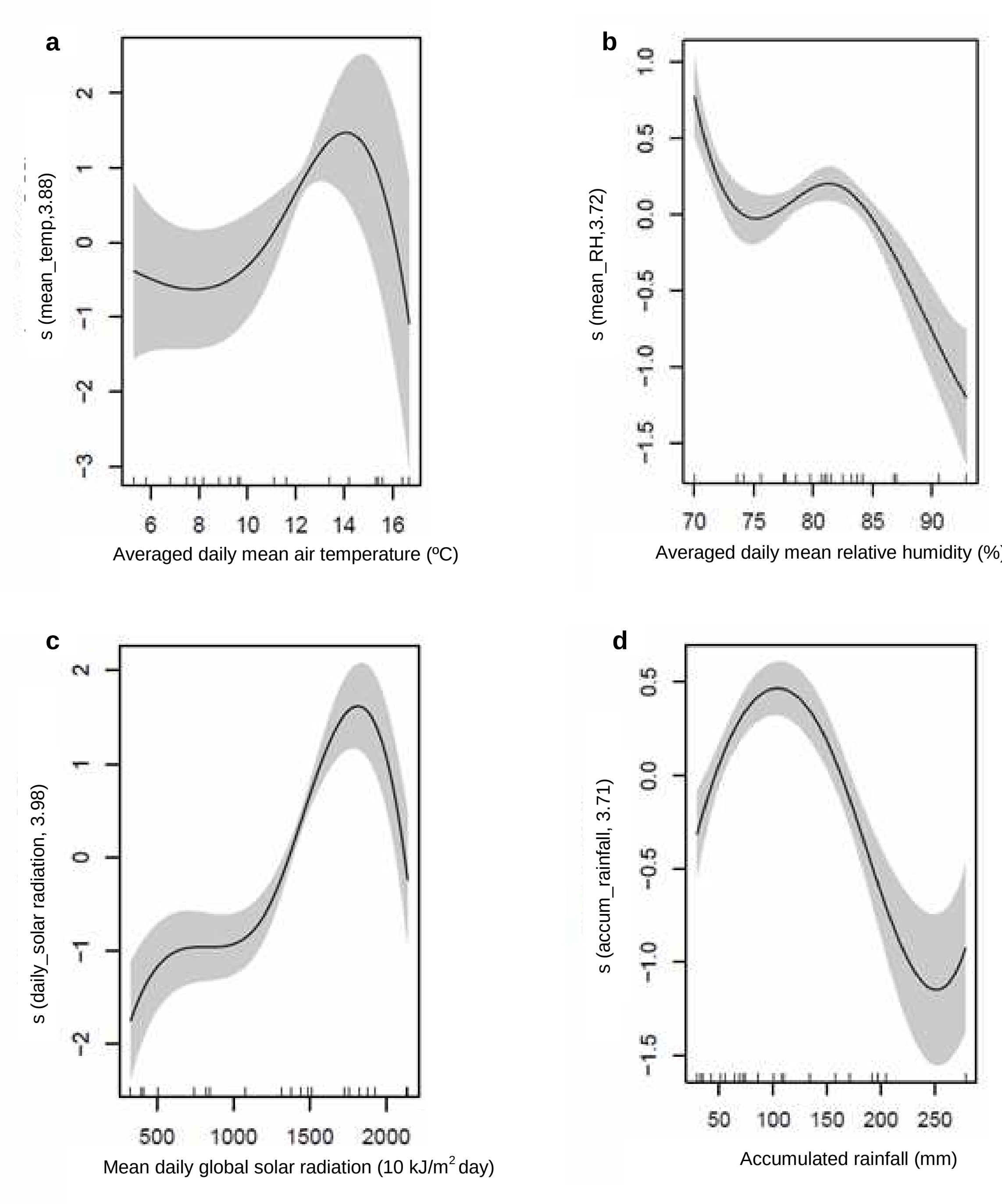

Figure S4 Relationships between the abundance of snails infected with *C. daubneyi* and averaged daily mean air temperature (a), averaged daily mean relative humidity (b), mean daily global solar radiation (c) and accumulated rainfall (d). Numbers on the *y* axis indicate the edf (effective degrees of freedom) for each effect. Shading indicates the 95% confidence bands.

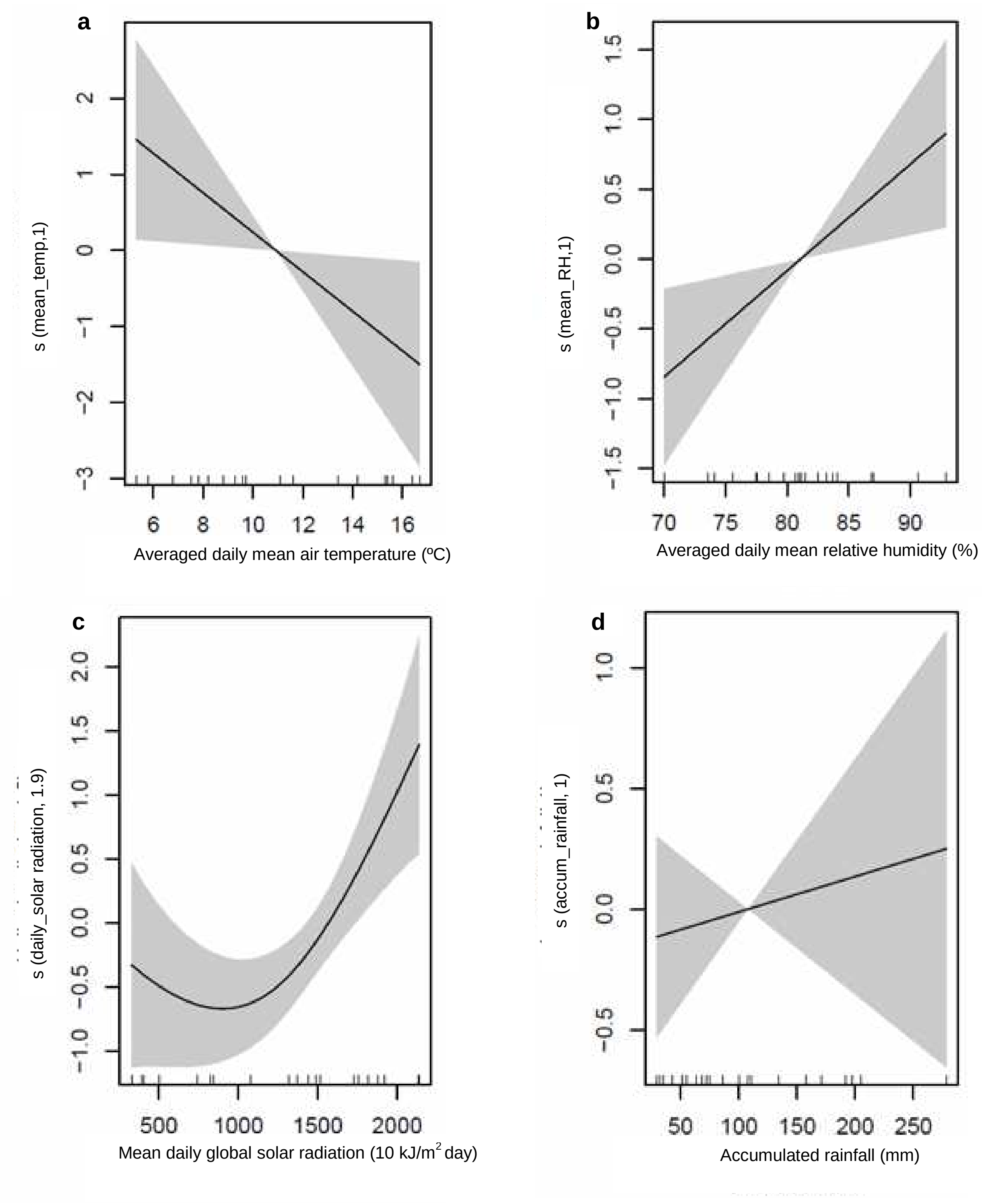

Figure S5 Relationships between the abundance of snails infected with *F. hepatica* and averaged daily mean air temperature (a), averaged daily mean relative humidity (b), mean daily global solar radiation (c) and accumulated rainfall (d). Numbers on the *y* axis indicate the effective degrees of freedom (edf) for each effect. Shading indicates the 95% confidence bands.

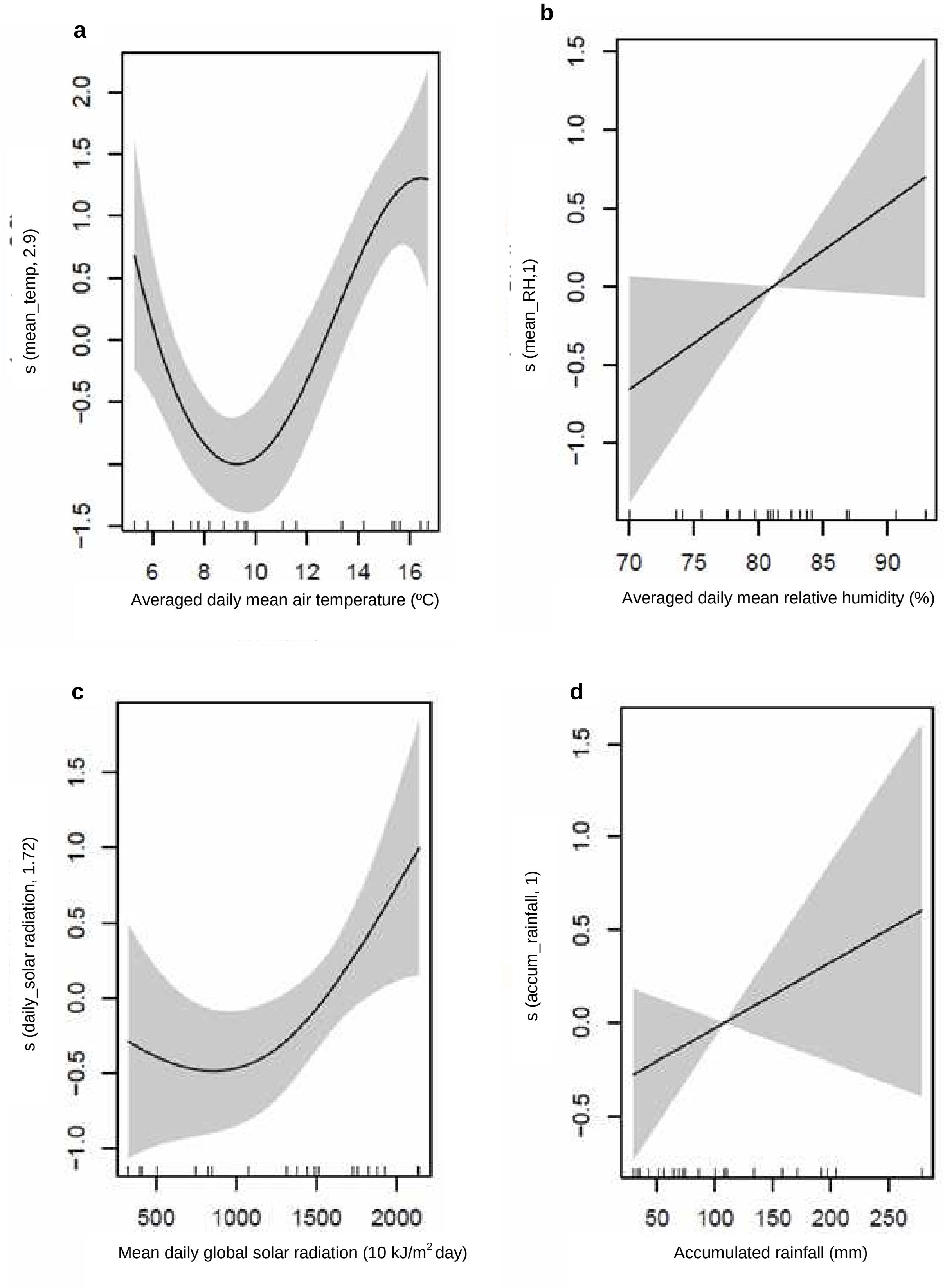

Figure S6 Temporal variation in the mean number of eggs per gram of faeces (epg) according to the centred smooth effect (s) estimated by the GAMs. *C. daubneyi*-epg (a), *F. hepatica*-epg (b). Numbers on the *y* axis indicate the effective degrees of freedom (edf) for each effect. Shading indicates the 95% confidence bands. Vertical lines separate seasons considered in the study period.

a

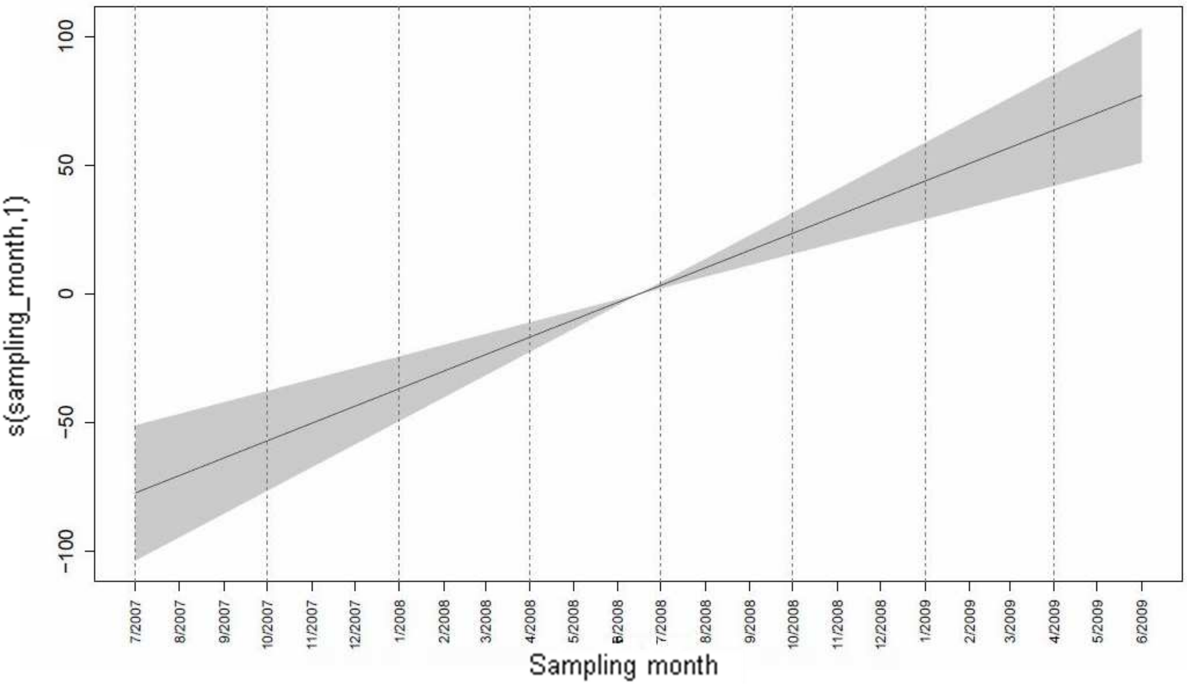

b

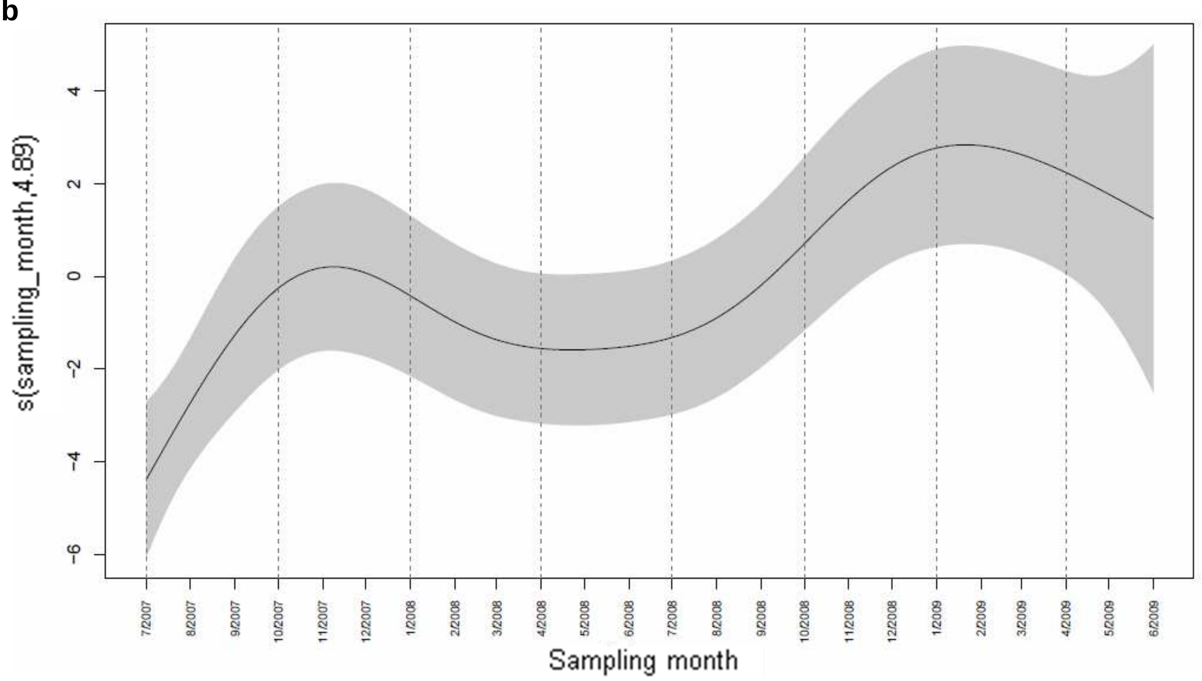

Figure S7 Monthly averaged values of daily mean air temperature (a) and daily global solar radiation (b), and monthly accumulated rainfall (c) recorded in the study area (Olas weather station) for the period July-2006 to June-2016.

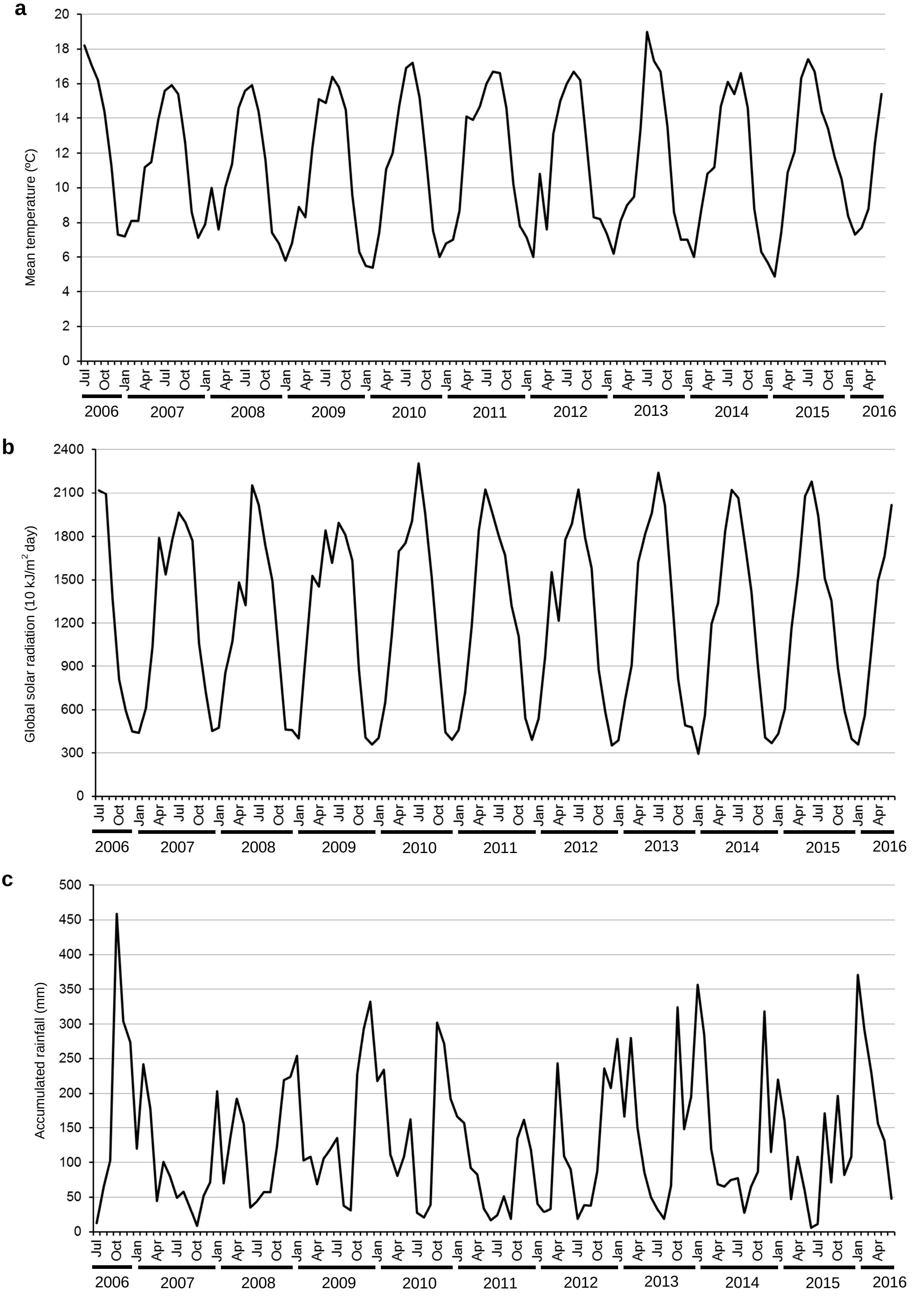

Supplement: Additional file 1: Figure S1. — Probability of infection with the trematodes C. daubneyi (a, b) and F. hepatica (c, d) in the snail G. truncatula, in relation to snail size (a, c) and sampling month (b, d), estimated by the smoothing effect(s) in GAMs. Figure S2. Temporal variation in snail populations according to the centred smoothing effect(s) in the GAMs: total number (a), numbers harbouring C. daubneyi (b) and F. hepatica (c). Figure S3. Relationships between snail abundance and averaged daily mean air temperature (a), averaged daily mean relative humidity (b), mean daily global solar radiation (c) and accumulated rainfall (d). Figure S4. Relationships between the abundance of snails infected with C. daubneyi and averaged daily mean air temperature (a), averaged daily mean relative humidity (b), mean daily global solar radiation (c) and accumulated rainfall (d). Figure S5. Relationship between the abundance of snails infected with F. hepatica and averaged daily mean air temperature (a), averaged daily mean relative humidity (b), mean daily global solar radiation (c) and accumulated rainfall (d). Figure S6. Temporal variation in the mean number of eggs per gram of faeces (epg) according to the centred smooth effect(s) estimated by the GAMs. Figure S7. Monthly averaged values of daily mean air temperature (a) and daily global solar radiation (b), and monthly accumulated rainfall (c) recorded in the study area (Olas weather station) for the period July 2006 to June 2016. (PDF 1987 kb) [file 13071_2016_1892_MOESM1_ESM.pdf]
